# The Breeding, Cultivation, and Potential Applications of Ornamental Orchids with a Focus on *Phalaenopsis*—A Brief Review

**DOI:** 10.3390/plants14111689

**Published:** 2025-05-31

**Authors:** Chenjing Han, Fei Dong, Yu Qi, Yenan Wang, Jiao Zhu, Binghai Li, Lijuan Zhang, Xiaohui Lv, Jianghui Wang

**Affiliations:** 1 Institute of Leisure Agriculture, Shandong Academy of Agricultural Sciences, Jinan 250100, China; hanchenjing0916@163.com (C.H.);; 2 Dongying Shuangfu Fusheng Agricultural Development Co., Ltd., Dongying 257086, China; 3 Horticultural Institute, Ningxia Academy of Agriculture and Forestry Sciences, Yinchuan 750002, China

**Keywords:** *Phalaenopsis*, breeding innovations, cultivation management, potential applications, prospects

## Abstract

The *Phalaenopsis* genus, a horticulturally vital group within the *Orchidaceae*, dominates global floriculture markets through strategic cultivar innovation, scalable propagation, and data-driven cultivation. This review systematically examines the breeding, propagation, cultivation management, and potential applications of *Phalaenopsis* while providing insights into future research directions. The main contents include the following: Breeding innovations—This review outlines the taxonomy of the *Phalaenopsis* genus and highlights its intergeneric hybridization potential, which offers vast opportunities for developing novel horticultural varieties. By establishing clear breeding objectives, researchers employ diverse breeding strategies, including conventional crossbreeding and biotechnological approaches (e.g., mutation breeding, ploidy manipulation, genetic transformation, and CRISPR/Cas9 editing). Propagation and cultivation management—Analyses of *Phalaenopsis* tissue culture protocols covering explant selection, media optimization, and regeneration systems are summarized. Key factors for efficient cultivation are discussed, including temperature, light, water, nutrient management, cultivation medium selection, and integrated pest/disease management. Scientific environmental control ensures robust plant growth, synchronized flowering, and high-quality flower production. Emerging applications—*Phalaenopsis* exhibits promising applications in functional bioactive compound extraction (e.g., antioxidants and antimicrobial agents). This review summarizes current advancements in *Phalaenopsis* breeding, cultivation, and potential applications. Based on technological progress and market demands, future research directions are proposed to support the sustainable development of the *Phalaenopsis* industry.

## 1. Introduction

As one of the largest angiosperm families, the *Orchidaceae* comprises over 750 genera and 31,485 documented species [1,2,3]. Orchids are renowned for their extraordinary diversity and vibrant colors, captivating observers with their stunning array of hues and intricate inflorescences [4]. Their extended shelf life further enhances their appeal, solidifying their status as a key commodity in the floral industry [5,6]. The *Phalaenopsis* genus stands out as one of the most horticulturally significant groups within the *Orchidaceae* family, being widely regarded as the top ornamental orchid globally [7]. These plants are highly sought after as both cut and potted flowers, making them a major commodity in international trade [8,9].

Due to their low hybridization barriers and distinctive reproductive mechanisms [9], the *Orchidaceae* family exhibits a remarkable ability to hybridize. Since 2003, approximately 500 new species within this family have been identified each year [10,11]. *Phalaenopsis* is among the most extensively researched genera, with significant advancements in breeding studies [12,13]. Currently, it includes 210 species, comprising 104 accepted names, 96 synonyms, and 10 unverified classifications [1]. Additionally, the Royal Horticultural Society (RHS) International Orchid Register Database records over 39,000 artificially created *Phalaenopsis* hybrids [14].

Over 70% of orchid species thrive as epiphytes [15], while others are terrestrial and require support for growth. Most orchids are perennial plants lacking permanent woody structures and display two growth patterns: monopodial and sympodial [16]. Both species and hybrids of *Phalaenopsis* are epiphytic and monopodial orchids [9,17]. Their leaves are thick and fleshy, with efficient water and nutrient storage, and possess crassulacean acid metabolism (CAM), enabling them to adapt to a wide range of environments [18].

**Figure 1:**
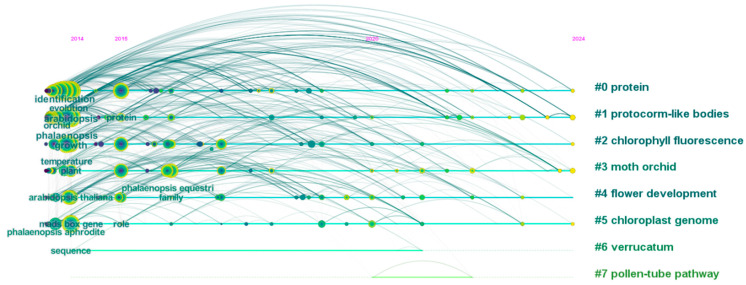
A temporal evolution visualization of keyword frequencies in *Phalaenopsis* research (2014–2024). The co-occurrence network was generated from articles indexed in Web of Science Core Collection using “*Phalaenopsis*” as the search term (2014–2024 publications) and analyzed through CiteSpace software (version 6.2.R4).

In the modern era, advancements in biotechnological interventions and cultivation techniques have significantly driven the growth of the orchid industry and its global trade [19]. The orchid industry not only generates sustainable outcomes but also establishes a high-value system. For instance, in 2020, the *Phalaenopsis* orchid market in the Netherlands was valued at EUR 422 million, with 117 million units sold [9]. The successful commercialization of *Phalaenopsis* orchids is closely tied to the development of new cultivars, efficient propagation systems, and intensive cultivation practices (Figure 1).

The orchid industry still faces critical scientific challenges, including the molecular mechanisms underlying *Phalaenopsis* floral fragrance, pigmentation, and stress responses under adverse conditions, as well as technical bottlenecks such as difficulties in gene editing delivery, innovative flower color breeding, the development of high-quality cultivation protocols, and product commercialization. This article provides an overview of orchid breeding, with a specific focus on *Phalaenopsis*, covering its classification and breeding methods. It also explores current and future trends in the development of cultivars, cultivation practices, and potential application.

## 2. Orchid Breeding

Orchids are primarily found in the tropical regions stretching from Asia to Australia [16]. Morphologically and anatomically, the *Orchidaceae* family can be divided into 5 subfamilies: *Apostasioideae* (2 genera and 16 species), *Cypripedioideae* (5 genera and 130 species), *Vanilloideae* (15 genera and 180 species), *Orchidoideae* (208 genera and 3630 species), and *Epidendroideae* (over 500 genera and 20,000 species) [20,21,22]. Among these, *Epidendroideae* is the largest subfamily [23,24]. Orchids are among the most hybridized, captivating, and commercially significant ornamental plants in global floriculture [9], with the *Phalaenopsis* genus, along with its intergeneric and interspecific hybrids, dominating as key cut and potted flowers in international flower markets. Therefore, understanding the classification of the *Phalaenopsis* genus is crucial for *Phalaenopsis* breeding.

### 2.1. The Classification of the Phalaenopsis Genus

The *Phalaenopsis* genus, commonly known as the moth orchid, is part of the Vandaeae tribe within the *Epidendroideae* subfamily [17,25]. Its flowers are characterized by three outer sepals, two petals, and a distinct lip petal, with the carpel positioned centrally and the stigma and stamen being fused into a single column. Despite its widespread popularity, the taxonomy of *Phalaenopsis* remains unclear, and its classification has been a subject of uncertainty for nearly two centuries [7,25].

In 1753, *Phalaenopsis* was first named by Linnaeus and classified under *Epidendrum* as ‘Epidendrum amabile’ [26]. In 1814, Roxburgh reclassified it into *Cymbidium* and named it ‘*Cymbidium amabilis*’ [7]. The genus *Phalaenopsis* was formally established by Blume in 1825, who renamed it ‘*Phalaenopsis amabilis*’ [7,26]. In 1980, Sweet categorized 46 species within the *Phalaenopsis* genus into nine sections, *Phalaenopsis*, *Proboscidioides*, *Aphyllae*, *Parishianae*, *Polychilos*, *Stauroglottis*, *Fuscatae*, *Amboinenses*, and *Zebrinae* [27], a classification that became foundational for subsequent taxonomic studies. In 2001, Christenson, based on morphological characteristics, divided approximately 66 species into five subgenera: *Proboscidioides*, *Aphyllae*, *Parishianae*, *Polychilos*, and *Phalaenopsis* [27,28]. This system remains the most authoritative morphological classification and is widely used in horticultural applications. Moving forward, the application of molecular marker technology to orchid classification and phylogenetic analysis will likely lead to a re-evaluation and discussion of previous classification systems [29].

### 2.2. The Breeding of Phalaenopsis

**Figure 2:**
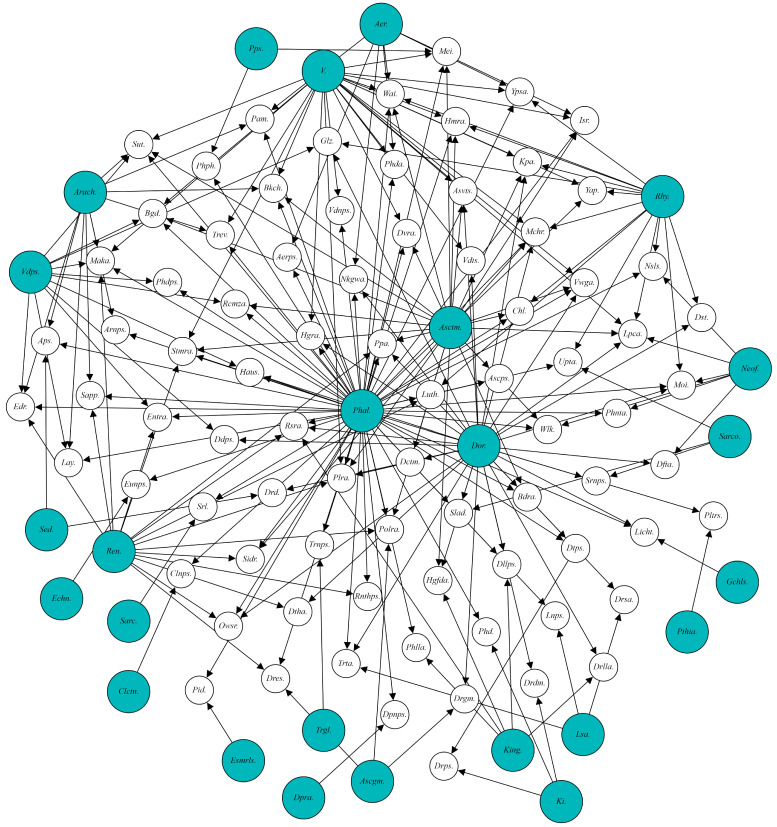
A visualization of *Phalaenopsis* species (green circles) and *Phalaenopsis* hybrids (white circles) created using NRD Studio software (Version 3.3.1). These hybrids were derived from crossbreeding through interspecific and intergeneric (Table S1, data source: Ichihashi and Mii [28]).

In horticulture, the term ‘moth orchid’ extends beyond just *Phalaenopsis* species and their interspecific hybrids [28]. While, taxonomically, closely related species are more likely to produce hybrids, the focus in horticulture and commercial breeding is on creating new hybrid plants [28]. This means that the commercial classification of orchids must be distinguished from their botanical classification [30]. The existence of natural hybrids in *Orchidaceae* demonstrates that many genetic barriers between species or genera are not fully established as criteria for reproductive incompatibility [28]. Most commercial orchid flowers are derived from interspecific or intergeneric hybrids. Over 20 closely related genera can hybridize with *Phalaenopsis*, enabling the development of numerous new hybrid genera and species [28] (Figure 2). Consequently, the potential to create innovative horticultural cultivars is immense.

#### 2.2.1. Innovation in New Horticultural Cultivars

The breeding of new horticultural cultivars focuses on a variety of desirable traits, including flower color, size, shape, texture, longevity, and resistance to pathogens [28]. For example, blue flowers have always been highly sought after, with the development of blue *Phalaenopsis* orchids being a significant milestone. Mii successfully created the world’s first blue *Phalaenopsis*, representing a breakthrough in orchid breeding [31]. Additionally, there has been growing market demand for mini-sized *Phalaenopsis* orchids, which are ideal for use as table flowers. Several mini cultivars have been developed, such as ‘KS Little Gem’, ‘Queen Beer’, ‘Tony Pink’, ‘Vaviche’, and ‘Rorens’, with the petal lengths ranging from 24.9 to 33.8 mm and the widths from 15.9 to 22.6 mm [32]. Among these, the mini-sized *P.* ‘Sogo Vivien’ stands out for its abundance of beautiful pink flowers [33], while ‘Bravo Star’ is prized for its fragrant, miniature pink blooms [34].

In terms of petal texture, *Phalaenopsis* flowers exhibit two distinct natural textures: velvety and waxy [35]. Velvety petals are larger, thinner, and lighter, offering striking visual appeal. A notable example is *P.* Sogo Yukidian ‘V3’, a renowned cultivar with large, white velvety petals [36,37]. On the other hand, waxy petals are thicker, more durable, and longer-lasting, making them suitable for long-distance transport. ‘Frigdaas Oxford’ is a medium–small cultivar with a yellow ground color and red-purple patterns, featuring waxy petals. It is also known for its high heat tolerance and resistance to pathogens [35,38]. These advancements highlight the diverse and innovative breeding efforts in the development of *Phalaenopsis* cultivars.

#### 2.2.2. Breeding Methods

Both conventional methods (e.g., selective breeding, hybridization) and biotechnological approaches, including haploid induction, polyploidization, genetic transformation, and CRISPR/Cas9 genome editing, are utilized to engineer flowers exhibiting diverse ornamental traits such as novel pigmentation, extended shelf life, and enhanced stress tolerance (Table 1). Despite technological advancements, conventional hybridization remains the dominant and most cost-effective strategy for generating new *Phalaenopsis* cultivars due to its high compatibility with interspecific crosses and predictable phenotypic outcomes [9,39].

##### Hybridization Breeding

Traditional cross-hybridization remains the most widely used conventional breeding method in *Phalaenopsis* [64]. Hybrid breeding serves as the foundational approach for combining different genotypes, whether that be within the same cultivar, between different varieties, or even across different species and genera [42,65]. The selection of parent plants is critical and depends on the specific breeding objectives. For instance, most deep red large-flowered *Phalaenopsis* orchids trace their lineage to *Doritaenosis* hybrids. A notable example is the cultivar *Dtps*. Hinacity Glow, which was developed by crossing *Dtps.* Coral Gleam and *P.* Herbert Hager [66]. Similarly, yellow *Phalaenopsis* cultivars are predominantly derived from species in the subgenus *Polychilos*, such as *P. amboinensis*, *P. fasciata*, *P. lueddemanniana*, and *P. venosa* [67]. The renowned yellow cultivar *P.* Taipei Gold, for example, is a hybrid of *P.* Glays Read ‘Snow Queen’ and *P. venosa* [68].

Spotted *Phalaenopsis* orchids often inherit traits from *P. lueddemanniana*. A landmark in *Phalaenopsis* breeding is *P.* Paifang’s Queen, which was created by crossing *P.* Mount Kaala and *P. lueddemanniana*. Its exceptional individual, *P.* Paifang’s Queen ‘Brother’, has been extensively used as a parent to breed red-spotted *Phalaenopsis* varieties [69]. Other key parents for spotted types include *P.* Golden Sands, *P.* Stuartiana, *P.* Ho’s Francy Leopard, *P.* Golden Peoker, *P.* Sentra, and *P.* Super Stupid [68,69].

White-colored *Phalaenopsis* orchids are also highly popular. The primary native species contributing to white flowers are *P. amabilis*, *P. aphrodite*, and *P. amabilis* var. *formosana* [70,71]. Additionally, other outstanding parents for breeding white *Phalaenopsis* include *P.* Chieftain, *P.* Joseph Hampton, *P.* San Marino, and *P.* Barbara Kirch [66,69]. These examples highlight the strategic use of hybridization to achieve diverse and desirable traits in *Phalaenopsis* breeding.

The above guidelines pertain to parental selection for breeding *Phalaenopsis* cultivars with diverse floral pigmentation. Additionally, the following principles are typically applied: maternal parent, prioritizing plants exhibiting vigorous growth and stable phenotypic performance; paternal parent, selecting individuals producing abundant, viable pollen with high germination rates (>85%); and trait-directed selection, when specific genetic traits (e.g., striped tepals) require maternal inheritance dominance. The parent carrying these target characteristics is designated as the maternal line [28].

##### Mutagenesis Breeding

Mutagenesis breeding encompasses both naturally occurring mutations and artificially induced mutations through the application of specific chemical agents or physical mutagens [72]. Artificial methods are particularly effective in increasing the frequency of mutations in plants [46,72]. Among these, gamma irradiation is a widely used technique [73,74]. Studies have shown that lower doses of gamma irradiation (around 15 Gy) can induce early flowering in *Phalaenopsis* [49,75], while higher doses (above 40 Gy) tend to inhibit flowering [76]. For instance, the *P. equestris* irradiated with a dose of 40 Gy exhibited rapid growth and development within just ten days post treatment [77]. Additionally, gamma-irradiated protocorms of *Phalaenopsis amabilis* have been used to develop mutants with enhanced resistance to soft rot disease [47]. These findings highlight the potential of mutation breeding, particularly gamma irradiation, in improving *Phalaenopsis* traits such as flowering time, growth rate, and disease resistance.

##### Ploidy Breeding

Ploidy breeding is a pivotal technique in plant genetics and involves the manipulation of chromosome numbers through the loss or gain of chromosomes relative to the normal chromosome complement [78]. This approach encompasses both haploid and polyploid breeding strategies. Haploid breeding, typically utilizing another culture technology, facilitates the rapid development of pure diploid lines through chromosome doubling, thereby circumventing the extensive processes of separation, selection, and stabilization inherent in conventional hybrid breeding. This method significantly accelerates the breeding cycle [79]. Moreover, haploid mutations are expressed directly without allelic interference, rendering their callus tissues optimal for mutation breeding. Artificial selection at the cellular level of callus tissue proves to be markedly more efficient than selection at the individual level [80]. In addition, there have been other attempts to induce haploids; for instance, Kazumitsu et al. explored the induction of haploid moth orchids by cultivating pseudofertilized ovules [81]. Similarly, Teixeira da Silva et al. attempted in vitro organogenesis in various parts of immature and fully opened *P.* Gallant Beau ‘George Vazquez’ flowers to generate haploid organs [82]. Although these efforts were not successful, they underscored the potential for such regeneration techniques in haploid production.

Polyploid breeding, involving the doubling of chromosomes through natural or artificial induction, is a crucial method for introducing genetic variation [83]. Polyploid cultivars often exhibit superior vigor, growth, stem sturdiness, leaf and flower size, and resistance compared to their diploid counterparts [84]. The *Phalaenopsis* genus primarily consists of diploid wild species, with a basic chromosome number of 2n = 2x = 38 [85,86]. However, the chromosome numbers of cultivated varieties vary, including tetraploid, triploid, and aneuploid forms [87], with tetraploid cultivars being predominant in commercial markets [88]. The ploidy differences between wild species and commercial cultivars complicate the transfer of desirable genes. Additionally, significant variations in chromosome size among *Phalaenopsis* species often result in the infertility of interspecific hybrids [89].

Polyploid induction in *Phalaenopsis* is typically achieved through chemical agents such as colchicine, oryzalin, trifluralin, and nitrous oxide (N_2_O) [54,89,90]. *Phalaenopsis amabilis* is the most commonly used species for such inductions [85,91,92]. Other varieties have also been tested for in vitro polyploid induction, including *P. amboinensis* [92], *P. equestris* [93], *P. bellina* [94], *P. aphrodite* [95], and various hybrid orchids (*Phalaenopsis* spp. and *Doritaenopsis* sp.) [96]. Common induction materials include protocorms and protocorm-like bodies (PLBs) [94], with unreduced gametes [85] and buds [97] also being utilized.

In summary, through both haploid and polyploid strategies, ploidy breeding offers significant advancements in plant breeding by accelerating the development of new varieties and enhancing desirable traits. The application of these techniques in *Phalaenopsis* orchids highlights their potential in overcoming breeding challenges and improving commercial cultivars.

##### Genetic Transformation and CRISPR/Cas9 Genome Editing Technology

Genetic transformation is a powerful technique for introducing exogenous genes or DNA fragments into plant genomes, enabling the transfer of traits that are unattainable through conventional hybridization [9]. In *Phalaenopsis*, genetic transformation can be achieved through particle bombardment and *Agrobacterium*-mediated methods, both of which are recognized as efficient and reliable approaches [6,98].

Particle bombardment is a direct physical transformation process and has been effectively utilized in *Phalaenopsis*. For instance, Chew et al. established an efficient particle bombardment transformation system for *P. bellina* using PLBs as target tissues [57]. Similarly, Fan employed particle bombardment to generate *P. equestris* var. alba plants resistant to *odontoglossum ringspot virus* (ORSV) by overexpressing either sense or anti-sense strands of the ORSV coat protein gene [99]. Additionally, Anzai et al. successfully delivered marker genes encoding *Escherichia coli β*-glucuronidase and *Aequorea victoria* green fluorescent protein into *Phalaenopsis* cells using this method [100].

In contrast, the *Agrobacterium*-mediated transformation system is often preferred due to its simplicity [9]. Belarmino et al. successfully produced transgenic *Phalaenopsis* plantlets by transforming suspension culture cells using *Agrobacterium tumefaciens* strains LBA4404 (Ptok233) and EHA101 (Pig121Hm) [101]. Semiarti et al. developed a genetic transformation method for *P. amabilis* using protocorms as the target material, mediated by *Agrobacterium tumefaciens* [102]. They further improved the transformation efficiency by pre-culturing protocorms on a medium containing an extract from fully ripe tomato fruit [103]. In a subsequent study, they utilized pollen (pollinia and pollinaria) of *P. amabilis* as target materials, immersing the pollen in an overnight culture of *Agrobacterium* and self-pollinating the flowers with the inoculated pollen. This approach allowed for the selection of positive transformants from the next generation, bypassing the need for in vitro inoculation [104].

The CRISPR/Cas9 system is a groundbreaking genome editing tool and enables precise modifications of target genes through guided sequence recognition [105]. Nopitasari et al. developed a method for delivering T-DNA into *P. amabilis* protocorms using *Agrobacterium tumefaciens* EHA101 carrying a T-DNA construct with UBI::Cas9::U3::VAR2 in the pRGEB32 vector [61]. To refine molecular breeding methodologies using CRISPR/Cas9, Semiarti et al. sowed *P. amabilis* seeds on NP+ peptone media (2 g L^−1^) to obtain protocorms, which were then submerged in a culture of *Agrobacterium tumefaciens* containing a T-DNA construct of the pRGEB32 vector harboring sgRNA with the *Phytoene desaturase* (*PDS*3) sequence [63]. Xia et al. advanced the field by developing two multiplex genome editing tools for *Phalaenopsis*: the PTG-Cas9-HPG (polycistronic tRNA-gRNA) system and the RMC-Cpf1-HPG (ribozyme-based multi-crRNA) system [60]. Furthermore, the CRISPR/Cas9 system has been employed to accelerate flowering in *P. amabilis* by inactivating the *Gibberellic acid insensitive* (*GAI*) gene, a mutation that enhances the regulation of *FLOWERING TIME* (*FT*) genes in the flowering biosynthesis pathway [59].

In summary, genetic transformation and genome editing technologies, including particle bombardment, *Agrobacterium*-mediated transformation, and CRISPR/Cas9, have significantly advanced the breeding and genetic improvement of *Phalaenopsis*, offering precise and efficient methods for trait manipulation and accelerated breeding cycles. After successfully breeding a new *Phalaenopsis* variety, the next steps involve expanding the population of superior cultivars, providing optimal cultivation environments and management practices, and ultimately cultivating robust plants that produce high-quality blooms.

## 3. Phalaenopsis Orchid Propagation and Cultivation Practices

### 3.1. The Propagation of Phalaenopsis

In their natural state, *Phalaenopsis* orchids primarily propagate through seeds, and artificial aseptic sowing can yield a large number of sterile seedlings [28]. However, seed propagation fails to meet the demands of factory production in terms of cultivar consistency and quantity [106]. Additionally, seedlings propagated through vegetative methods often exhibit inconsistent characteristics [107]. Therefore, tissue culture has become the primary approach for mass-producing elite commercial *Phalaenopsis* cultivars [108].

A variety of explants can be used for *Phalaenopsis* tissue culture, including flower stalks [109,110,111], shoot tips [112,113,114], leaves [107,110,115], and roots [116,117]. Among these, flower stalks are the most commonly selected explants. The Murashige and Skoog (MS) culture medium is the most widely used for *Phalaenopsis* propagation.

For instance, Chen employed a root medium composed of 1/2 MS nutrients supplemented with 200 mL L^−1^ coconut liquid, 6.5 g L^−1^ agar, 10 g L^−1^ sucrose, 10 mg L^−1^ 6-benzylaminopurine (BA), and 5.0 mg L^−1^ α-naphthaleneacetic acid (NAA) to cultivate *P.* Sogo Yukidian ‘V3’ [118]. This study also evaluated the effects of temperature and light irradiance on growth characteristics. Similarly, Minh utilized an MS culture medium enriched with BA, thidiazuron (TDZ), β-indol butyric acid (IBA), NAA, adenine, 6-furfurylaminopurine, 10 mg L^−1^ vitamin B_1_, 1 g L^−1^ peptone, 10% coconut water, 30 g L^−1^ sucrose, and 1 g L^−1^ activated carbon to investigate the large-scale propagation of *Phalaenopsis* orchids using bioreactor technology [115]. Furthermore, Barough et al. evaluated the effects of eight different culture media on the growth of in vitro mini plantlets of *Phalaenopsis* orchids. Among the eight culture media tested, the SM2 high-carbon formulation demonstrated the highest growth rate. Its balanced 20-20-20 NPK ratio mitigated the inhibitory effects of high nitrogen levels on seed germination while promoting leaf and root development to enhance regeneration efficiency. SM2’s replacement of synthetic vitamins (e.g., nicotinic acid, thiamine) with banana powder and other natural components reduced chemical mutagenesis risks, supporting genetic stability. When combined with the TIS-FA-Bio system, SM2 balanced the growth rate, regeneration capacity, and genetic stability while achieving a 72.5% cost reduction [119]. Their findings highlighted that the optimal culture medium for orchid growth is highly dependent on the specific cultivation system employed.

Tissue culture, particularly the use of flower stalks as explants and MS-based media, has become the cornerstone of *Phalaenopsis* propagation, enabling the production of consistent and high-quality cultivars for commercial purposes. Advances in culture media formulations and bioreactor technology further enhance the efficiency and scalability of this process.

### 3.2. Cultivation Practices of Phalaenopsis Orchid

#### 3.2.1. The Biological Characteristics of Phalaenopsis

*Phalaenopsis* orchids exhibit unique biological traits that enable them to thrive in their natural environments. They possess short stems and thick, succulent leaves with stomata that open at night, an adaptation that minimizes water loss in water-limited conditions. This characteristic allows them to perform CAM photosynthesis, which enhances water use efficiency and maximizes CO_2_ uptake [120].

Native to subtropical and tropical regions, *Phalaenopsis* orchids are adapted to consistently warm temperatures [121]. Consequently, they are highly sensitive to low temperatures and susceptible to cold injury [122]. Temperature plays a critical role in their flowering process, with floral induction being inhibited when temperatures exceed 28 °C [123].

In their natural habitat, *Phalaenopsis* orchids grow epiphytically on trees, with their roots exposed to moist air [124]. This adaptation makes them vulnerable to both drought and waterlogging. Drought conditions cause leaves to become wrinkled and flaccid, while waterlogging damages the roots.

The leaves of *Phalaenopsis* orchids naturally droop, and the amount of light they receive is influenced by tree shade and the angle of light incidence. This light intensity is optimal for their growth [28]. Under typical temperature and low-light conditions, *Phalaenopsis* orchids can maintain a long flower display life [124].

The biological characteristics of *Phalaenopsis* orchids, including their CAM photosynthesis, temperature sensitivity, epiphytic growth habit, and light requirements, are key to their survival and ornamental value. Understanding these traits is essential for their successful cultivation and conservation.

#### 3.2.2. Environmental Adaptability of Phalaenopsis Cultivars

The environmental adaptability of *Phalaenopsis* cultivars is a critical factor in their successful cultivation. For instance, cultivars bred in cooler regions often struggle to adapt to high-temperature summer conditions [28]. In areas with prolonged and intense summer heat, *Phalaenopsis* cultivation necessitates the use of cooling systems such as fans and water curtains, resulting in significant energy consumption [125]. Therefore, selecting cultivars with superior heat resistance is essential. *P.* Tongzhen, for example, exhibits the highest heat tolerance and is particularly suitable for cultivation in regions with hot summers, such as southern China [126].

Conversely, in low-temperature winter regions, heating systems are required to maintain optimal growing conditions, which not only increases costs but also leads to substantial energy consumption. Cold-tolerant cultivars, however, can thrive in relatively low temperatures without the need for excessive energy input [127]. Several *Phalaenopsis* cultivars are known for their strong low-temperature tolerance, including *P. mannii*, *P. lobbi*, *P.* Alishan, *P.* In the Mood for Love, *P.* Wedding Promenade, *P.* Love at First Sight, and *P.* Fule Star. These cultivars are well suited for cultivation in middle- to high-latitude regions [128,129].

Whether for heat tolerance in warm climates or cold tolerance in cooler regions, the selection of *Phalaenopsis* cultivars with appropriate environmental adaptability can significantly reduce energy consumption and operational costs, ensuring sustainable and efficient cultivation practices.

#### 3.2.3. Environmental Factors of Phalaenopsis Cultivation

To obtain high-quality *Phalaenopsis* orchids, it is essential to provide a suitable environment and implement appropriate cultivation management practices. Below are key factors to consider (Table 2).

##### Temperature

*Phalaenopsis* orchids, like many tropical plants, are sensitive to cold temperatures. Prolonged exposure to temperatures below 15 °C can lead to physiological issues. Daems et al. investigated the effects of chilling temperatures on the photosynthetic performance of *P.* ‘Edessa’ and found that leaf photosynthesis was impaired after 24 h at 10 °C, with a significant decline in the performance index [130]. Similarly, Ma et al. studied *P. amabilis* ‘Green Bear’ and *P. amabilis* ‘Anna’ at 5 °C for 12, 24, and 48 h. They observed that chilling stress caused frozen spots on new leaves and the wilting of old leaves by the second day, with severe damage and leaf drop by the fourth day in *P. amabilis* ‘Anna’ [143]. Mu et al. suggested that spraying *Phalaenopsis* leaves with Sodium Nitroprusside solution could mitigate low-temperature stress [144].

For optimal growth and flowering, *Phalaenopsis* orchids are typically cultivated in greenhouses with controlled temperatures. High temperatures are maintained during the growth phase, while lower temperatures are necessary for inflorescence initiation [123,145]. Newton and Runkle emphasized that a daytime temperature of 26 °C or lower is crucial for inflorescence initiation, while exposure to temperatures above 29 °C for 8 or 12 h can inhibit flowering [146]. Jeong et al. found that high temperatures before the induction phase delayed flowering initiation and inflorescence development in *P.* Queen Beer ‘Mantefon’ [131]. Lee et al. demonstrated that intermittent high temperatures could prevent premature flowering by reducing the percentage of visible inflorescences and delaying their emergence while also decreasing soluble sugar content in leaves [123,147].

Temperature management is critical for the healthy growth and flowering of *Phalaenopsis* orchids. Both excessively low and high temperatures can disrupt physiological processes, emphasizing the need for precise temperature control in cultivation practices.

##### Water

Proper watering is essential for maintaining the health and vitality of *Phalaenopsis* orchids. When adequately watered, the relative water content of their leaves remains between 85 and 94%, and the roots exhibit a vibrant green color [134]. However, under drought stress, the roots take on a silvery hue, signaling water deficiency [124]. Tay et al. demonstrated that after 7 weeks of drought, *P. cornu-cervi* experienced a significant reduction in photosynthetic light utilization, with the relative water content in leaves dropping below 50% [134]. Ceusters et al. investigated the effects of drought stress on the photosynthetic performance of *P.* ‘Edessa’. Their study revealed that drought conditions led to increased thermal dissipation and the inactivation of partial PS‖ reaction centers, disrupting the electron flow from PS‖ to PS| [133]. Jeong and Oh examined the impact of 40 days of simulated shipping without watering on the flowering of *P.* Sogo Yukidian ‘V3’ [132]. Their findings indicated that drought-stressed plants experienced delayed flowering. To mitigate such stress, Gu proposed the application of chitosan spray on leaves, which was shown to alleviate drought stress in *Phalaenopsis* seedlings by enhancing the content of osmoregulatory substances [148].

Maintaining optimal watering practices is crucial for ensuring the photosynthetic efficiency, root health, and timely flowering of *Phalaenopsis* orchids. Drought stress not only impairs physiological functions but also delays developmental processes, underscoring the importance of consistent and adequate water management in cultivation.

##### Light

Light plays a pivotal role in photosynthesis and the overall health of *Phalaenopsis* orchids. However, excessive light exposure can lead to the formation of brown spots on the leaves, adversely affecting growth [135]. In computer-controlled greenhouses, the optimal light intensity for *Phalaenopsis* cultivation typically ranges between 200 and 300 µmol m^−2^ s^−1^ [149].

Ko et al. demonstrated that acclimating young tissue-cultured seedlings and 2.5′′ potted plants to blue light prior to transplantation significantly reduced photoinhibition, enhancing their adaptability to greenhouse conditions [137]. Magar et al. investigated the influence of light quality on *Phalaenopsis* flowering and discovered that red light markedly promoted flowering, increasing the number of florets per stem by 33.8% compared to blue light [136]. In a subsequent study, Magar et al. reaffirmed that red light was the most effective in stimulating flowering, whereas green light had the least impact. Far-red light exhibited effects similar to those of blue light [150].

Managing light intensity and quality is crucial for optimizing the growth and flowering of *Phalaenopsis* orchids. While excessive light can cause damage, appropriate light conditions, particularly the use of red light, may significantly enhance flowering performance.

##### Fertilization

While potted *Phalaenopsis* orchids can initially derive nutrients from the cultivation medium, essential fertility tends to leach away with each watering. Thus, maintaining a balanced and consistent supply of all necessary nutrients is critical for optimal growth and flowering [28]. Fertilization guidelines for *Phalaenopsis* vary, reflecting diverse approaches to nutrient management.

Poole and Seeley proposed an optimal fertilization regimen during the nutrient culture stage, consisting of nitrogen (N), potassium (K), and magnesium (Mg) at 100 ppm, 50–100 ppm, and 25 ppm, respectively [151]. Wang and Chang emphasized the importance of maintaining a steady supply of N throughout both the vegetative and reproductive phases to maximize growth and flowering potential. They also highlighted that *Phalaenopsis* orchids prefer nitrogen in the nitrate (NO_3_^−^) form, recommending NO_3_^−^ as the primary N source [152].

In a recent study, Alves et al. explored the effects of calcium (Ca) fertilization on *Phalaenopsis* cultivation. Their results demonstrated that increasing Ca concentrations up to 6.25 mM significantly enhanced plant length, flower quality, and leaf texture. Additionally, higher Ca levels were correlated with increased N and phosphorus (P) contents in the substrate, further supporting plant health and development [153].

Effective fertilization practices for *Phalaenopsis* orchids require a balanced supply of essential nutrients, particularly nitrogen in the nitrate form, alongside potassium, magnesium, and calcium. Tailoring fertilization strategies to the specific growth stages and nutrient preferences of *Phalaenopsis* can significantly improve plant vigor, flower quality, and overall cultivation success.

##### Cultivation Medium

The cultivation medium is a critical component for orchid growth, providing anchorage, retaining moisture and nutrients, and ensuring proper root aeration. Commonly used media for orchid cultivation include tree bark, coconut husk chips, fir cocopeat, and sphagnum moss. Among these, sphagnum moss is the most widely used medium for *Phalaenopsis* orchids due to its superior performance.

Kaveriamma et al. compared the effects of coconut chips, cocopeat, and sphagnum moss on *P.* ‘Magic Kiss’. Their findings revealed that plants cultivated in sphagnum moss exhibited significantly better vegetative and floral characteristics compared to those grown in coconut husk chips or coconut husk bits. Specifically, *Phalaenopsis* orchids grown in sphagnum moss achieved greater height, with an average increase of 1.64 cm versus 0.21 cm in cocopeat and 0.40 cm in coconut husk chips. Moreover, inflorescence emergence occurred earliest in sphagnum moss (124.90 days) compared to 153.30 days in coconut husk chips and 155.90 days in cocopeat [141].

However, sphagnum moss is a natural resource and its extensive harvesting has led to increased market prices and concerns about depletion due to limited supply. This has spurred the development of alternative cultivation media. For instance, peanut shells have been explored as a viable substitute. Research by Hanik et al. demonstrated that peanut shell-based media performed comparably to fern-based media across key metrics, including leaf count, leaf width, root count, and average fresh weight [142]. This suggests that peanut shell mixed media can serve as a sustainable and effective alternative to traditional substrates.

Additionally, innovative approaches such as the use of artificial textile fibers have been investigated as cultivation substrates for *Phalaenopsis* orchids [154]. Furthermore, studies in closed plant factories have explored the cultivation of young *Phalaenopsis* plants without potting media, utilizing nutrient solutions and rhizosphere ventilation [155]. These alternative methods represent promising advancements in orchid cultivation, offering sustainable and efficient solutions for the future.

While sphagnum moss remains the preferred medium for *Phalaenopsis* cultivation due to its superior performance, the development of alternative substrates such as peanut shells and artificial fibers, as well as innovative soilless cultivation techniques, provides sustainable options to address resource limitations and environmental concerns.

##### Pests and Diseases

*Phalaenopsis* orchids are predominantly cultivated in greenhouses, where high humidity and elevated temperatures create favorable conditions for the proliferation of various pests and diseases. Kim et al. conducted a comprehensive two-year study on pest species affecting the above-ground parts of *Phalaenopsis* orchids across nine farms in South Korea. They identified a total of 10 insect species, with *Tenuipalpus pacificus* Baker and *Frankliniella intonsa* Trybom emerging as the primary pests impacting orchid health [156]. In Guangdong, China, *Bradysia difformis* Frey has been recognized as a significant pest, causing severe damage to seedlings in greenhouse environments [157]. Additionally, *Dichromothrips corbetti*, commonly known as Vanda thrips, has become a critical pest, posing substantial risks to the global commercial cultivation of *Phalaenopsis* orchids, particularly in large-scale production systems [158].

Among viral pathogens, *Cymbidium mosaic virus* (CymMV) and ORSV are the most widespread and economically significant, affecting *Phalaenopsis* orchids on a global scale [5]. Consequently, extensive research has been conducted on virus detection [159,160], virus–host interactions [161,162], and control measures [163,164]. Furthermore, the development of transgenic lines with dual resistance has been explored [165]. Recent studies have increasingly focused on the development of genetically modified orchids engineered for enhanced resistance to viral infections [11].

The cultivation of *Phalaenopsis* orchids in greenhouse environments is susceptible to a range of pests and diseases, including insect pests as well as viral pathogens like CymMV and ORSV. Ongoing research in virus detection, host interactions, control measures, and genetic engineering is crucial for mitigating these threats and ensuring the sustainable production of *Phalaenopsis* orchids.

The successful cultivation of *Phalaenopsis* orchids requires the careful management of temperature, water, light, fertilization, cultivation medium, and pests and diseases. Maintaining optimal conditions ensures healthy growth, timely flowering, and high-quality blooms.

## 4. The Potential Applications of Phalaenopsis

Beyond their widespread use as potted plants and cut flowers, *Phalaenopsis* orchids offer a range of promising applications, particularly in the fields of antioxidants, antimicrobials, and cosmetics. Among these, their antioxidant activity has been the most extensively studied (Table 3).

Minh et al. identified that ethyl acetate extracts derived from the roots of hybrid *Phalaenopsis* spp. exhibited the highest level of antioxidant activity, suggesting that these root extracts could serve as an effective source of natural antioxidants [166,171]. Nguyen et al. further explored the antioxidant potential and secondary metabolite compounds in methanol extracts from four parts (roots, leaves, flower stalks, and flowers) of three differently colored orchids: white, yellow, and purple [167]. Their findings revealed that the leaf extract of the white orchid demonstrated the highest efficacy in the DPPH radical scavenging assay, while the flower extract of the white orchid exhibited the strongest reducing power.

Irimescu et al. investigated extracts derived from *Phalaenopsis* waste materials, such as dried leaves, stems, and roots [168]. Their results indicated that leaf and root extracts exhibited moderate to high antioxidant potential, with the leaf extract showing significant inhibitory activity against methicillin-resistant *Staphylococcus aureus* (MRSA) and *Pseudomonas aeruginosa*. The stem extract also demonstrated inhibitory activity against *Bacillus cereus*. Yamada et al. proposed that *Phalaenopsis* orchid extract (Phex) inhibits the differentiation of melanocyte stem cells, suggesting its potential as a novel therapeutic agent for treating solar lentigines [172]. These findings highlight the potential of *Phalaenopsis* extracts in the pharmaceutical and cosmetic industries [173].

In addition to antioxidant activity, the antimicrobial properties of *Phalaenopsis* extracts have also been investigated. Studies have shown that extracts derived from PLBs of *Phalaenopsis* significantly inhibit the growth of *Acidovorax citrulli*, reduce bacterial attachment to watermelon seeds, and mitigate disease symptoms in infected watermelon seedlings. Researchers hypothesize that the reprogramming of cellular activity during the PLB regeneration process generates metabolites with potent antibacterial properties [169]. Tzou et al. demonstrated that mixed liposomes formed from monogalactosyldiacylglycerol (MGDG) glycolipids in *Phalaenopsis* leaves significantly enhance the suppression of *Escherichia coli* growth during both the growth and spike induction phases [174].

Chiu et al. examined the petals and sepals of nine moth orchid varieties with distinct colors and identified significant quantities of two C-glycosylated flavones, reaching up to 1.5% and 5.6% on a dry weight basis. These findings suggest that moth orchid flowers could serve as a valuable resource for the exploration of both known and yet-to-be-discovered applications [170].

*Phalaenopsis* orchids hold immense potential beyond their ornamental value, with applications in antioxidants, antimicrobials, and cosmetics. Their extracts demonstrate significant biological activities, paving the way for future innovations in pharmaceuticals, agriculture, and skincare industries.

## 5. Conclusions and Prospects

Currently, conventional breeding techniques remain the most widely used method for *Phalaenopsis* orchid breeding, yielding the majority of commercial varieties. Intergeneric hybridization also offers significant potential for creating novel orchid cultivars, expanding the diversity and unique characteristics of these plants [9]. Genetic transformation technology enables the development of varieties with desirable traits such as novel flower colors, unique flower shapes, and enhanced stress resistance, providing precise and effective methods to achieve specific breeding goals [175]. This technology is poised to become a powerful tool in *Phalaenopsis* orchid breeding, particularly for overcoming challenges such as breeding red orchids and developing pure true red *Phalaenopsis* varieties [176].

*Phalaenopsis* orchids are renowned for their distinctive fragrance and are highly prized in the ornamental flower market [177]. The harmonious blend of scent and visual beauty offers consumers a multi-sensory experience, while the fragrance itself evokes pleasant emotions, significantly enhancing the emotional allure of these orchids. Due to their unique qualities, fragrant *Phalaenopsis* orchids are particularly sought after in the premium floral market. With their distinctive sensory experience and emotional value, large-flowered and fragrant *Phalaenopsis* orchids are recognized as a key focus in *Phalaenopsis* breeding [178,179].

Resource-efficient *Phalaenopsis* orchid varieties also represent a crucial direction in breeding programs. In some regions where *Phalaenopsis* orchids are cultivated on a large-scale, year-round *Phalaenopsis* production incurs high energy costs due to strict temperature control requirements [180]. For instance, flower induction in summer demands nighttime temperatures of 18 °C and daytime temperatures of 25 °C [125,181], while winter cultivation requires nighttime temperatures no lower than 18 °C [182]. Therefore, breeding high-temperature flowering varieties capable of spike initiation and flowering at 20–25 °C alongside cold-tolerant varieties that withstand low temperatures of 15 °C during winter production can substantially reduce energy consumption. In natural conditions, *Phalaenopsis* orchids require approximately 3 years from seed germination to flowering [9]. Under tissue culture propagation, this timeline can be reduced to at least 18 months by extending photoperiods and implementing precise water and nutrient management. Breeding fast-growing *Phalaenopsis* varieties can further shorten cultivation cycles and reduce energy demands in production systems. Additionally, *Phalaenopsis* orchids often require staking during cultivation due to the gravitational stress exerted by their heavy multi-flowered inflorescences. Breeding programs targeting enhanced stem lignification and thus producing self-supporting flower spikes without artificial stabilization represent a strategic approach to streamline production costs in commercial orchid operations [183].

In *Phalaenopsis* cultivation, the development of a balanced nutrient formula specifically tailored for these orchids remains an urgent priority. The implementation of a showerhead-style downward irrigation system for fertilizer delivery effectively prevents the localized accumulation or deficiency of specific nutrients in the growing substrate. The integration of nanobubble technology with a balanced nutrient formulation can enhance absorption efficiency, thereby optimizing resource utilization and minimizing environmental pollution [184,185].

In terms of potential applications, Rizky et al. developed a micropropagation protocol utilizing natural compounds, enabling the cultivation of unique ‘orchid key holder’ prototypes in miniature glass vessels [186]. This innovation aims to advance agritourism through horticultural biotechnology. The growing global economy and expanding cultural prosperity have driven the increased consumer demand for cultural creative products, which are emerging as a pivotal economic force [187]. The development of *Phalaenopsis*-themed cultural creative products has garnered increasing attention as a niche market segment, synergizing botanical esthetics with cultural narratives to bridge horticultural innovation and consumer-driven value creation [188].

**Figure 3:**
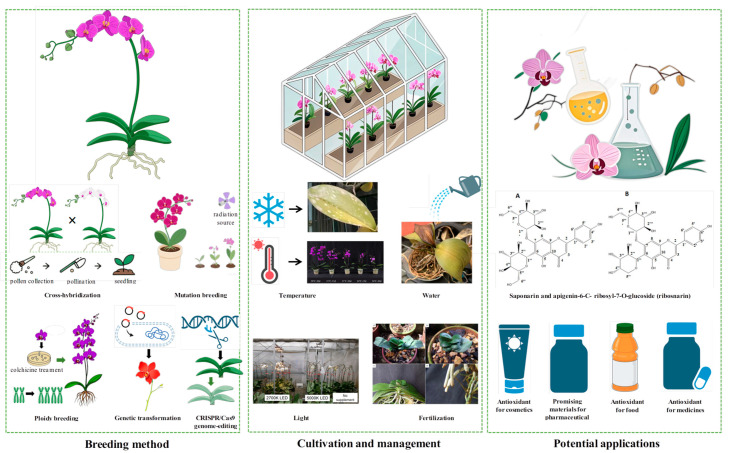
Integrated framework of *Phalaenopsis* breeding, cultivation management, and potential applications. Icons were created using BioRender and AI-Doubao (https://www.doubao.com/chat/). All referenced images are credited below. In terms of ‘cultivation and management’, image data about ‘high temperature’ are sourced from Jeong et al. [131], image data about ‘light’ are sourced from Magar et al. [136], and image data about ‘fertilization’ are sourced from Novais et al. [140]. In terms of ‘potential applications’, image data about ‘Saponarin and apigenin-6-C-ribosyl-7-O-glucoside (ribosnarin)’ are sourced from Chiu et al. [170].

In conclusion, the future of *Phalaenopsis* breeding and cultivation lies in leveraging advanced technologies, optimizing resource efficiency, and exploring innovative applications (Figure 3). By integrating conventional breeding, genetic transformation, and sustainable cultivation practices, the *Phalaenopsis* industry can continue to thrive, meeting consumer demands while minimizing environmental impact.

## Figures and Tables

**Table 1:** Breeding methods for *Phalaenopsis* cultivars.

Method	Process Description	Results	Reference
Cross-hybridization	Hybridization between *P. pulcherrima* fma. *coerulea* and *Van*. Lilac Blossom, with resulting hybrids serving as bridge parents.	Successfully bred an intergeneric hybrid *Yap*. Tariflor Pink Fairy ‘Tainung No. 2-Pink Fairy’.	[40]
	Introducing harlequin type to *P. pulcherrima* through cross breeding between *P.* I Hsin Bee x *P. pulcherrima* var. *champorensis*.	35% of offspring expressed both dark pigmentation and spotted patterning inherited from the maternal parent.	[41]
	Using SSR and SCoT marker systems to investigate cross ability of *V. stangeana* and *P. hygrochila.*	Hybridization of two different genera can successfully produce an intergeneric hybrid.	[42]
	Using *P.* Fire Cracker as female parent and *Rhy*. *coelestis* as male parent to breed blue *Phalaenopsis* hybrids.	Successfully bred deep and nonfading blue color *Phalaenopsis* hybrid: *Rhy*. Tariflor Blue Kid ‘1030-4’.	[43]
	Analyzing chromosomal compositions of 60 representative *Phalaenopsis* cultivars.	Genome size of parent species varies, and gene infiltration of parent species are also different.	[44]
	Using *P.* ‘KS Little Gem’ and *P.* ‘1747’ as parental lines to perform reciprocal crosses.	Selection of ‘KS Little Gem’ as female parent during hybridization can produce longer shelf-life *Phalaenopsis*.	[12]
	Pollinating *P.* Sunrise Goldmour ‘KHM637’ by removing upper column section while preserving intact stigma cavity.	Overcame cross barriers by cut-column pollination method and successfully obtaining offsprings.	[17]
Mutagenesis breeding	Using ^60^Co-γ-ray with dose of 40 Gy to treat *P. equestris*.	Increased leaf growth, sugar content, and antioxidant ability.	[45]
	Observing morphological variations in M1V0 generation of *P. amboinensis* irradiated with gamma-rays at different doses.	Successfully induced morphological diversity in *P. amboinensis* var.	[46]
	Using γ-ray ranging from 5 to 20 Gy to irradiate protocorms to develop soft-rot resistance *Phalaenopsis* mutants.	75% disease-resistant *Phalaenopsis* regenerants were derived from protocorm explants subjected to 5 Gy γ-ray exposure.	[47]
	Using fast neutron impulse pile to irradiate *Phalaenopsis* PLBs and seedling stem segments.	Enhanced multiplication and differentiation of PLBs and promoted seedling stem segment multiplication.	[48]
	Using γ-ray ranging from 0 to 25 Gy to irradiate invitro plantlets of *P.* amabilis in order to cultivate early-flowering *Phalaenopsis*.	Early-flowering mutants emerged in 25 Gy treatment group, achieving blooming within 13 months post acclimatization.	[49]
Ploidy breeding	Using colchicine concentrations of 0, 0.01%, 0.05%, and 0.10% to treat *Phalaenopsis* flower buds at meiotic stage for 3 d.	0.05% colchicine applied during leptotene–zygotene stage for 3 d, increasing 2n pollen frequency to 10.04%.	[50]
	Using colchicine concentrations of 0.05%, 0.10%, and 0.2% to treat PLBs of *Phalaenopsis* H-03 (2n = 2x = 38) and co-culture for 5, 10, 15 d.	Applyied 0.05% colchicine for 15 d, resulting in approximately 30% plant survival and 50% mutation rates.	[51]
	Complete plantlets developed from PLB cultures underwent 72 h immersion in an oxygenated bioreactor with colchicine.	Highest tetraploid induction success rate was achieved with 0.15% colchicine, but only 50% survived.	[52]
	*Phalaenopsis* protocorms were cultured in 5 pre-treatment media for 8 weeks. They were then soaked in 50 mg L^−1^ colchicine for 10 d.	Optimal pre-treatment is the combination of 15% CW and TDZ.	[53]
	Using colchicine concentrations ranging from 0 to 2000 mg L^−1^ to treat *Phalaenopsis* flowers and development pods (fruit) for 3–5 d.	Application of 50 mg L^−1^ and 500 mg L^−1^ resulted in 60% and 100% tetraploid seedlings, respectively.	[54]
Genetic transformation	Investigating the influence of varying injection sites, acetosyringone concentrations, and injection volumes on *P. amabilis*.	Optimal conditions: abaxial side of leaf, 200 µM acetosyringone, and injection volume of 500 µL.	[55]
	Using *Agrobacterium*-mediated transformation, *P.* Sogo Yukidian ‘SPM313’ cultivar was genetically modified.	Transgenic lines exhibited distinct phenotypic modifications including darker-green, wider and shorter leaves, etc.	[56]
	Using PLBs from *P. bellina* as target tissues, particle bombardment parameters and biological parameters were optimized.	Optimal distance of 6 cm, helium pressure of 1100 psi, gold particle size of 1.0 μm, vacuum pressure of 27 mmHg.	[57]
	T-DNA vector containing an eGFP marker was introduced via *Agrobacterium tumefaciens* EHA105-mediated transformation.	Stable transformation system for *Phalaenopsis* was established. Transformation efficiency reached approximately 1.2–5.2%.	[8]
	T-DNA containing 35S::GAL4::AtRKD4::GR construct was inserted into *P.* ‘Sogo Vivien’ PLBs.	17 transgenic plants carrying *AtRKD*4 and *HPT* genes were successfully obtained, with transformation efficiency of 0.63%.	[58]
CRISPR/Cas9 genome editing	Analyzing protein domain of *GAI* (Gibberellic Acid Insensitive) in *P. amabilis*, and designing a single guide RNA.	*GAI* gene possesses unique amino acid sequences and domains, making it ideal target for CRISPR/Cas9 gene editing.	[59]
	Developing two multiplex genome editing tools for *Phalaenopsis* genome editing.	Both PTG-Cas9-HPG and RMC-Cpf1-HPG multiplex genome editing systems are functional in *Phalaenopsis* orchids.	[60]
	Vector containing the CRISPR/Cas9 system and hygromycin phosphotransferase (HygR) was delivered into *P. amabilis* PLBs.	Leaf color of transformed plants changed from green to yellowish-green or yellow.	[61]
	2 strategies were employed for multi-gene mutagenesis in *Phalaenopsis equestris*: 3sg1C strategy and 3X1sg strategy.	3sg1C strategy: high editing efficiency, 97.9%. 3X1sg strategy: single-gene editing, most transformants carried only one sgRNA.	[62]
	Vector containing *HygR* and the Cas9-sgRNA system was employed to target two sites (*PDS*3*T*1 and *PDS*3*T*2) of *PDS3* gene.	Transformation efficiencies of *PDS*3*T*1 and *PDS*3*T*2 were 0.9% and 0.96%, respectively. Leaves exhibit albino phenotype.	[63]

**Table 2:** The cultivation and management of *Phalaenopsis*.

Factors	Treatments	Results	References
Temperature	*P.* ‘Edessa’ was exposed to a low temperature at 10 °C for 3 weeks. Photosynthetic performance and metabolic responses were evaluated.	The electron transport chain from PSII to PSI was impaired. Rubisco activity declined significantly. Carbon metabolism was imbalanced.	[130]
	Four *Phalaenopsis* cultivars were exposed to intermittent high-temperature. Vegetative growth and flowering parameters were measured.	Intermittent high-temperature treatment reduced the flowering rate by 40–60%.	[123]
	*P.* Queen Beer ‘Mantefon’ were exposed to 28 °C (control), 31 and 34 °C for 15/30 d prior to the induction phase at 20 °C.	Under 34 °C, 30 d, visible inflorescence emergence was prolonged by 22 d (54%), and the total flowering period extended up to 191.7 d.	[131]
Water	*P.* Sogo Yukidian ‘V3’ were treated in simulated shipping conditions with light (LSS) and dark (DSS) treatments without irrigation.	DSS without irrigation for 40 d resulted in a sharp increase in leaf-yellowing rate (16.9%) and failed to recover to the normal.	[132]
	Treatment group, complete water cessation (drought stress simulation); control group, twice-weekly irrigation.	The performance index significantly decreased, accompanied by imbalance between light energy absorption and utilization.	[133]
	Physiological responses of *P. cornu-cervi* under varying light and water conditions were measured.	After 7 weeks of drought treatment, Fv/Fm and leaf RWC significantly decreased, and proline increased.	[134]
Light	*Phalaenopsis* were treated with two shading intensities (60% and 70%) and four fertilizer concentrations (1, 2, 3, and 4 g L^−1^).	For 60% shade, 2 g L^−1^ promotes leaf expansion and root elongation, while 70% shade requires 4 g L^−1^ to compensate for light limitations.	[135]
	*P. amabilis* and *P.* Sogo Yukidian ‘V3’ were treated with a supplemental light.	Supplemental lighting significantly promoted flowering and flower scape growth.	[136]
	*P. aphrodite* subsp. *Formosana* seedlings and mature plants were pre-treated with blue light for 12 days.	After blue light acclimation, *Phalaenopsis* exposed to high light exhibited a higher Fv/Fm and electron transport rate.	[137]
Fertilization	The effects of four new controlled-release fertilizers (NCRFs) with different release rates on *Phalaenopsis* growth were tested.	When NCRFs were 1.5 g/pot, the highest values for leaf length, leaf width, fresh weight, and root weight were obtained.	[138]
	Four nitrogen concentration treatments (0 × N, 0.1 × N, 1 × N, and 2 × N) were applied from the late vegetative stage through the reproductive stage.	High nitrogen (28.6 mM) caused an 8.9 d spiking delay in *P.* ‘F2510’. Low nitrogen (1.4 mM) or 0 × N advanced spiking by 1–2 d.	[139]
	The phosphorus–zinc interaction effects on dry matter accumulation and P, Zn, Fe, and Mn uptake in *Phalaenopsis* orchids were explored.	Increasing P rates reduced root dry weight. High P significantly suppressed Zn translocation.	[140]
Cultivation medium	The effects of sphagnum moss versus coconut husk chips and cocopeat on growth parameters of *P.* ‘Magic Kiss’ were compared.	Plants in sphagnum moss exhibited earlier inflorescences, a longer inflorescence length, and an extended flowering duration.	[141]
	Comparing the effects of peanut shell medium, mixed medium (fern + peanut shell), and pure fern medium on *Phalaenopsis* orchid growth.	No significant differences were found in *Phalaenopsis* orchid growth. The peanut shell can effectively replace fern medium.	[142]

**Table 3:** The antioxidant activity of *Phalaenopsis*.

Materials	Extraction	Antioxidant Substances and Potential Applications	References
Leaves, stems, and roots of *P.* Sogo Yukidian ‘V3’	Samples were extracted with ethanol and solvents of varying polarities through stepwise extraction to obtain free phenolics.	Extracts from *Phalaenopsis* orchid roots (especially ethyl acetate) are a highly effective natural antioxidant source, with potential to replace synthetic antioxidants.	[166]
Roots, pedicels, leaves, and flowers of white (‘City More’), yellow (‘Sogo Meili’), and purple (‘Queen Beer’) *Phalaenopsis* orchids	In total, 0.25 g of dry powder of each plant part was immersed in 5 mL methanol at room temperature. The liquid phase was then separated from the cell debris through filtration under a vacuum using filter paper to obtain the crude orchid extract.	Extracts of white orchids showed the strongest activity in reducing power assays, while those of yellow and purple orchids exhibited the highest effectiveness in ferrous ion-chelating ability tests. These extracts serve as potential antioxidant sources with medicinal value and stress resistance.	[167]
Leaves, stems, and roots of *Phalaenopsis* orchid	The material was dried, ground, and extracted with 70% ethanol and methanol using a maceration method and then filtered to obtain crude extract.	Extracts significantly inhibit MRSA and *Pseudomonas aeruginosa*, and against *Bacillus cereus*. They can serve as natural alternatives against drug-resistant bacteria and be used in anti-aging skincare products or functional foods.	[168]
PLBs of *Phalaenopsis* orchid	The PLB extract was prepared through liquid nitrogen grinding, ethyl acetate ultrasonic extraction, and C18 column chromatography fractionation.	The PLB extract significantly reduced the growth rate of *A. citrulli.* Treated watermelon seeds showed reduced bacterial counts on their surfaces. The PLB extract could serve as a novel biopesticide for seed treatment or field disease control.	[169]
Flower components, including petals, sepals, and lip of *Phalaenopsis* orchid	Petals, sepals, and lips were separately cut into pieces and weighed. They were homogenized with 10 volumes of 60% methanol using a homogenizer and ultrasonicated for 10 min. The homogenate was centrifuged at 9000× *g* for 3 min. The supernatant was membrane-filtered (0.45 μm).	White *Phalaenopsis* orchid petals and sepals showed the highest content of saponarin and ribosnarin. They could serve as antioxidants or functional food additives. These compounds provide lead candidates for developing anti-inflammatory or anti-diabetic drugs.	[170]

## Data Availability

The data presented in this study are available on request from the corresponding author.

## Supplementary Materials

The following supporting information can be downloaded at https://www.mdpi.com/article/10.3390/plants14111689/s1, Table S1: A comprehensive list of species names along with their respective parent.
