# Supplementary material for: The Breeding, Cultivation, and Potential Applications of Ornamental Orchids with a Focus on Phalaenopsis—A Brief Review
Source: Plants (Basel). 2025 May 31;14(11):1689. doi: 10.3390/plants14111689 (PMC12157862; doi:10.3390/plants14111689)
Supplement: Supplementary file 1 [file plants-14-01689-s001.zip › plants-3636731-supplementary.pdf]

Supplementary Table S1. A comprehensive list of species names along with their respective parent.

(**data source:** Ichihashi and Mii [28])

**Genus**	**abbreviation**	**respective parent**
Aerides	*Aer.*	
Arachnis	*Arach.*	
Ascoglossum	*Ascgm.*	
Ascocentrum	*Adctm.*	
Cleisocentron	*Clctn.*	
Diploprora	*Dpra.*	
Doritis	*Dor.*	
Eurychone	*Echn.*	
Esmeralda	*Esmrls.*	
Gastrochilus	*Gchils.*	
Kingidium	*Ki.*	
Kingiella	*King.*	
Luisia	*Lsa.*	
Neofinetia	*Neof.*	
Paraphalaenopsis	*Pps.*	
Pelatantheria	*Pthia.*	
Phalaenopsis	*Phal.*	
Renanthera	*Ren.*	
Rhynchostylis	*Rhy.*	
Sarcanthopsis	*Sara.*	
Sarcochilus	*Sarco.*	
Sedirea	*Sed.*	
Tricoglottis	*Trgl.*	
Vanda	*V.*	
Vandopsis	*Vdps.*	

Aeridopsis	*Aerps.*	*Aer.× Phal.*
Arachnopsirea	*Aps.*	*Arach. × Phal. × Sed.*
Arachnopsis	*Arnps.*	*Arach. × Phal.*
Asconopsis	*Ascps.*	*Asctm.× Phal.*
Ascovandoritis	*Asvts.*	*Asctm.× Dor. ×V.*
Bearara	*Bdra.*	*Asctm.× Dor. × Phal.*
Bogardara	*Bgd.*	*Asctm.× Phal. × V. × Vdps.*
Bokchoonara	*Bkch.*	*Arach. × Asctm.× Phal. × V.*
Chinheongara	*Chl.*	*Asctm. × Phal. × Rhy.*
Cleisonopsis	*Clnps.*	*Clctn. × Phal.*
Devereuxara	*Dvra.*	*Asctm.× Phal. × V.*
Diplonopsis	*Dpnps.*	*Dpra. × Phal.*
Dorandopsis	*Ddps.*	*Dor. × Vdps.*
Doredirea	*Drd.*	*Dor. × Sed.*
Doricentrum	*Dctm.*	*Asctm. × Dor.*
doridium	*Drdm.*	*Dor. × Ki.*
doriella	*Drlla.*	*Dor. × King.*
doriellaopsis	*Dllps.*	*Dor. × King. × Phal.*
dorifinrtia	*Dfta.*	*Dor. × Neof.*
doriglossum	*Drgm.*	*Ascgm. × Dor.*
doriopsisium	*Drps.*	*Dtps. × Ki.*
dorisia	*Drsa.*	*Dor. × Lsa.*
doristylis	*Dst.*	*Dor. × Rhy.*
doritaenopsis	*Dtps.*	*Dor. × Phal.*
dorthera	*Dtha.*	*Dor. × Ren.*
dresslerara	*Dres.*	*Ascgm. × Phal. × Ren.*
edeara	*Edr.*	*Arach. × Phal. × Ren. × Vdps.*
ernestara	*Entra.*	*Phal. × Ren. × Vdps.*
eurynopsis	*Eunps.*	*Echn. × Phal.*

glanzara	*Glz.*	*Dor. × Rhy. × Vdps.*
hagerara	*Hgra.*	*Dor. × Phal. × V.*
hausermannara	*Haus.*	*Dor. × Phal. × Vdps.*
himoriara	*Hmra.*	*Asctm. × Phal. × Rhy. × V.*
hugofreedara	*Hgfda.*	*Asctm. × Dor. × King.*
isaoara	*Isr.*	*Aer. × Asctm. × Phal. × V.*
kippenara	*Kpa.*	*Asctm. × Dor. × Rhy. × V.*
Laipenchihara	*Lpca.*	*Asctm. × Dor. × Neof. × Rhy. × V.*
laycockara	*Lay.*	*Arach. × Phal. × Vdps.*
lichtara	*Licht.*	*Dor. × Gchls. × Phal.*
luinopsis	*Lnps.*	*Lsa. × Phal.*
lutherara	*Luth.*	*Phal. × Ren. × Rhy.*
macekara	*Maka.*	*Arach. × Phal. × Ren. × V. × Vdps.*
meechaiara	*Mchr.*	*Asctm. × Dor. × Phal. × Rhy. × V.*
meirmosesara	*Mei.*	*Asctm. × Pps. × Phal. × V.*
morieara	*Moi.*	*Dor. × Neof. × Phal. × Rhy.*
nakagawaara	*Nkgwa.*	*Aer. × Dor. × Phal.*
neostylopsis	*Nsls.*	*Neof. × Phal. × Rhy.*
owensara	*Owsr.*	*Dor. × Phal. × Ren.*
parnataara	*Pam.*	*Aer. × Arach. × Phal.*
paulara	*Plra.*	*Asctm. × Dor. × Phal. × Ren. × V.*
pelatoritis	*Pltrs.*	*Dor. × Pthia.*
pepeara	*Ppa.*	*Asctm. × Dor. × Phal. × Ren.*
phalaenidium	*Phd.*	*Ki. × Phal.*
phalaerianda	*Phda.*	*Aer. × Phal. × V.*
phalandopsis	*Phdps.*	*Phal. × Vdps.*
phalanetia	*Phnta.*	*Neof. × Phal.*
phaleralda	*Pid.*	*Esmrls. × Phal.*
phaliella	*Phlla.*	*King. × Phal.*

phalphalaenopsis	*Phph.*	*Pps.* × *Phal.*
pooleara	*Polra.*	*Asctm.* × *Ascgm.* × *Phal.* × *Ren.*
renanthopsis	*Rnthps.*	*Phal.* × *Ren.*
richardmizutaara	*Rcmza.*	*Asctm.* × *Phal.* × *Vdps.*
roseara	*Rsra.*	*Dor.* × *King.* × *Phal.* × *Ren.*
sappanara	*Sapp.*	*Arach.* × *Phal.* × *Ren.*
sarcalaenopsis	*Srl.*	*Phal.* × *Sarc.*
sarconopsis	*Srnps.*	*Phal.* × *Sarco.*
sidranara	*Sidr.*	*Asctm.* × *Phal.* × *Ren.*
sladeara	*Slad.*	*Dor.* × *Phal.* × *Sarco.*
stamariaara	*Stmra.*	*Asctm.* × *Phal.* × *Ren.* × *V.*
sutingara	*Sut.*	*Arach.* × *Asctm.* × *Phal.* × *V.* × *Vdps.*
trautara	*Trta.*	*Dor.* × *Lsa.* × *Phal.*
trevorara	*Trev.*	*Arach.* × *Phal.* × *V.*
trichonopsis	*Trnps.*	*Phal.* × *Trgl.*
uptonara	*Upta.*	*Phal.* × *Rhy.* × *Sarco.*
vandaenopsis	*Vdnps.*	*Phal.* × *V.*
vandewegheara	*Vwga.*	*Asctm.* × *Dor.* × *Phal.* × *V.*
vandoritis	*Vdts.*	*Dor.* × *V.*
waibengara	*Wai.*	*Aer.* × *Asctm.* × *Phal.* × *Rhy.* × *V.*
wikara	*Wlk.*	*Asctm.* × *Phal.* × *Neof.*
yapara	*Yap.*	*Phal.* × *Rhy.* × *V.*
yeepengara	*Ypsa.*	*Aer.* × *Phal.* × *Rhy.* × *V.*
